# The Role of the Ovary in the Induction of Tumours by the Local Application of 9,10-Dimethyl-1,2-Benzanthracene to the Genital Tract of Rats

**DOI:** 10.1038/bjc.1958.6

**Published:** 1958-03

**Authors:** A. Glucksmann, Cora P. Cherry

## Abstract

**Images:**


### THE ROLE OF THE OVARY IN THE INDUCTION OF TUMOURS BY THE LOCAL APPLICATION OF 9,10-DIMETHYL-1,2-BENZANTHRACENE TO THE GENITAL TRACT OF RATS

A. GLUCKSMANN AND CORA P. CHERRY*
*From the Strangeways Research Laboratory, Cambridge*

Received for publication November 12, 1957

THE incidence of cancer of the uterine cervix appears to be affected by hormonal factors and there is also some suggestion that the tumour type may be influenced by the endocrine state (Glucksmann and Cherry, 1956). Experimentally it has been possible to induce cancer of the uterine cervix by hormonal treatment as well as by hydrocarbons. The literature on this subject has been well reviewed by Gardner (1953) and von Haam and Scarpelli (1955). Most of the work has been done with mice in which with the exception of one strain (Gardner and Pan, 1948) the incidence of spontaneous cancers of the cervix is rare. The hydrocarbons proved more effective in tumour production than the oestrogens and the resulting tumours were mainly squamous-cell carcinomas. Sarcomas were rare and none are mentioned in von Haam's report of the literature nor by Reagan, Wentz and Machicao, (1955) and Murphy (1953). Gardner and Pan (1948) observed 1 sarcoma and 12 carcinomas in the PM strain of mice in which the tumour occurred " spontaneously ". In hybrids of that strain treated with oestrogen $\pm$ androgens, only carcinomas were found (Pan and Gardner, 1948*a*). On subcutaneous grafting of uterine cervices and cornua together with methylcholanthrene crystals, Pan and Gardner (1948*b*) obtained in strain A and hybrid mice almost as many sarcomas as carcinomas.

Rats have not been used extensively for these studies. McEuen obtained a few cancers of the uterine cervix after prolonged treatment with oestrogens. Pfeiffer (1949) reports 4 leiomyomas and an adenocarcinoma of the uterus in female rats in which testes had been grafted shortly after birth. Vellios and Griffin (1957) using a technique similar to that of Murphy (1953) and of Reagan *et al.* (1955) induced 13 tumours of the cervix in 39 rats in 28 weeks. One of these tumours was a carcinosarcoma, the others were carcinomas. Using spermicidal contraceptives applied per os or per vaginam daily for 14 to 18 months to rats kept on a low protein diet Hoch-Ligeti (1957) induced 21 tumours in 95 animals. These tumours are described as " fibrous or adenomatous polyps covered by multilayered squamous epithelium . . . Three tumours had characteristics of sarcomas, with invasion of the uterine wall, irregular mitoses, giant cells and marked anaplasia ". In addition (?) an angiofibrosarcoma and an epidermoid carcinoma are given as illustrations. The rats on the low protein diet showed liver damage which may have impaired the detoxication of oestrogens. It is not clear from the account how many of the tumours were sarcomas or carcinomas and what role, if any, interference with the oestrogen metabolism played in the induction of the uterine tumours.

* Working with a grant from the British Empire Cancer Campaign.

The experiments described in the present paper were undertaken to study the influence of endocrine factors on the incidence and type of tumours of the female genital tract ; 9,10-dimethyl-1,2-benzanthracene was applied locally to the vagina and vulva of virgin rats, of castrated rats and of castrated rats treated with oestradiol.

### MATERIALS AND METHODS

Female hooded rats were used throughout this study and their age at the beginning of the experiments was between 3 and 4 months. 9,10-Dimethyl-1,2-benzanthracene (DMBA) obtained from Messrs. L. Light and Co. was the carcinogenic agent and a 1 per cent solution in acetone was used.

Twenty-eight virgin animals were painted intravaginally once weekly with DMBA for 28 weeks and thereafter the painting was done twice weekly for a further 27 weeks after which the experiment ended. Twelve virgin rats, which served as controls, were painted with acetone once weekly for 28 weeks and thereafter twice weekly.

Forty rats were castrated between the ages of two and three months and were used for the experiments one to two months later. Sixteen of these castrated animals were painted intravaginally twice weekly with a 1 per cent solution of DMBA in acetone. Another 16 rats were treated as above and in addition were given twice weekly intramuscular injections of 1$\mu$g. oestradiol monobenzoate (Organon) in olive oil, the injections being given the day before the painting with the carcinogen. Eight castrated rats served as controls and were painted intravaginally twice weekly with acetone.

The cervix and vagina were painted by means of a cotton wool swab on the end of a galvanised wire. The vagina was stretched open by dorsal flexion of the tail, the swab was inserted and the cervix and vagina painted by means of a rotary motion. By this method of painting the vulva was contaminated with DMBA.

Animals were killed from 200 to 410 days after the beginning of the experiment and the uterus, cervix, vagina and vulval skin were removed and in the virgin rats the ovaries were also taken out. Six of the virgin rats and 3 of the castrated rats in the experimental groups had to be killed for other conditions before 200 days and they have not been included in the experimental results.

The tissues were fixed in Zenker's fluid or Susa solution and embedded in paraffin. Sections were cut at 8 $\mu$ and stained with haematoxylin and eosin, by the periodic acid-Schiff technique with and without diastase digestion, with Southgate's mucicarmine stain or with van Gieson's stain.

A quantitative analysis was made by cell counts and by measurements of the vaginal and vulval epithelium and of the stroma of the vagina and uterus in the various experimental and control groups. Straight regions of the epithelium without carcinomas or warts were selected, the height of the epithelium was measured and total cell counts were made over a unit length of 200 $\mu$. In the vagina the width of the stroma was taken from the lower edge of the epithelium to the inner muscle layer and in the uterus from the epithelium to the inner circular muscle layer.

### RESULTS

(a) *Acetone painting.*—The repeated intravaginal applications of acetone caused some thickening of the vaginal epithelium and marked cornification in both virgin and castrated rats. Keratohyaline granules were present in a well-developed stratum granulosum which was covered by fairly thick layers of keratin (Fig. 6). No effect of the acetone painting on the epithelium of the vulva could be detected (Fig. 1). Apart from occasional slight inflammatory infiltrations the vaginal stroma showed no abnormalities. None of the acetone-painted rats produced tumours in either the vagina or the skin at the introitus vaginae (vulva).

Fig. 1.—The average height ■ and the average number of cells □ per unit length of vulval epithelium. The standard deviations are indicated by lines in each column.

A.—Acetone treatment of castrated rats.
B.—DMBA treatment of castrated rats.
C.—DMBA + oestradiol treatment of castrated rats.
D.—Acetone treatment of virgin rats.
E.—DMBA treatment of virgin rats.

(b) *Castration.*—The castrated control rats painted with acetone showed an atrophy of the vaginal epithelium as compared with the acetone painted virgin rats. Measurements of the height of the epithelium (Fig. 2) indicate that the average thickness in the castrated animals was only 58 per cent of that in virgin rats. The total cell count per unit length of the epithelium was also reduced but only to 89 per cent. This disparity between the reduction in the height of the epithelium and in the total cell count can be attributed to a smaller average size of the epithelial cells in the castrates so that in a given volume of tissue more of the smaller cells were present than of the larger cells in the virgin rats.

The epithelium of the vulva in castrated and virgin rats painted with acetone showed the same thickness and total number of cells (Fig. 1) and is apparently not affected by castration.

The vaginal stroma of the castrated animals was thinner and less cellular than that of the virgin rats. Measurements show a relative reduction in width of the stroma by castration to 48 per cent (Fig. 3). An even greater reduction in width

FIG. 2.—The average height ■ and the average number of cells □ per unit length of vaginal epithelium. The standard deviations are indicated by lines in each column.

A.—Acetone treatment of castrated rats.
B.—DMBA treatment of castrated rats.
C.—DMBA + oestradiol treatment of castrated rats.
D.—Acetone treatment of virgin rats.
E.—DMBA treatment of virgin rats.

of the uterine stroma was observed after castration (Fig. 3), the width of the stroma in the castrates being only 40 per cent of that in virgin rats (Fig. 7–8). The oestrogen treatment restored the width of the vaginal and uterine stroma to that of the virgin rats (Fig. 9).

Castration thus decreases the volume of uterine stroma, vaginal stroma and vaginal epithelium in that order, but does not affect the epithelium of the vulva (Fig. 1).

(c) *DMBA painting on virgin rats* was followed by marked thickening of both vaginal and vulval epithelium. The vaginal epithelium showed marked cornification and had some downward projections. In only two animals did this hyperplasia proceed to tumour formation : one rat had a papilloma (Fig. 10) and another a squamous-cell carcinoma (Fig. 11) arising in the vaginal epithelium. Both these animals were killed 380 days after the beginning of treatment.

The hyperplasia of the vulval epithelium was much more striking than that of the vagina and was followed by the appearance of warts and of squamous-cell carcinomas in 95 per cent of the 22 animals at risk.

While the hyperplasia of the vagina caused an increase in the total number of cells to 124 per cent of that in the acetone-painted controls (Fig. 2) and an increase to 146 per cent in the height of the epithelium, the comparable figures for the

Fig. 3.—The average width of the stroma in the vagina ■ and in the uterus □. The standard deviations are indicated by lines in each column.

A.—Acetone treatment of castrated rats.
B.—DMBA treatment of castrated rats.
C.—DMBA + oestradiol treatment of castrated rats.
D.—Acetone treatment of virgin rats.
E.—DMBA treatment of virgin rats.

vulval hyperplasia are 325 per cent and 800 per cent respectively (Fig. 1). The far greater increase in width of the epithelium than in cell count is accounted for by changes in cell size.

The first tumour of the vulva was histologically identified as a squamous-cell carcinoma in a rat killed 253 days after the beginning of treatment. Of the 22 rats surviving for 250 or more days 21 had tumours at the vulva of which 15 were carcinomas and 6 were warts. The tumours were often multiple and large necessitating the killing of the animal. Apart from squamous-cell carcinomas some basal-cell carcinomas arising in the hair follicles were found. The papillomas tended to precede the appearance of carcinomas which often arose in them. Of the 6 warts, 4 were found in rats killed before the 350th day while of the 15 carcinomas only 7 were found before that time. The animals with warts were killed, because of tumours in the vagina, probably before the malignant change occurred.

Apart from the carcinomas of the vulva the most striking change produced by DMBA painting was the appearance of sarcomas in the stroma of the vagina. Of the 22 rats at risk 16 were found to have sarcomas of the vaginal wall. The first tumour was found after 283 days i.e. 30 days later than the first carcinoma of the vulva, but the subsequent appearance of tumours was very similar to that of carcinomas of the vulva : 8 appeared before the 350th day and the average time for sarcomas on histological verification was 333 days as against 332 days for the carcinomas of the vulva.

The sarcomas varied in extent from growths involving the vagina, uterus, bladder and other pelvic structures to microscopic lesions extending for only a few sections. In type they were mostly cellular fibrosarcomas containing numerous multinucleate giant cells (Fig. 12, 13). The smallest lesions arose in perivascular infiltrations in the vaginal stroma in which the normal fibrous structure had disappeared, and the cellular infiltrate was characterised by the appearance of large cells with prominent nuclei containing coarse chromatin granules (Fig. 14, 15). These cells were found in varying proportions in the fully developed and invasive sarcomas (Fig. 13). Some of the smaller lesions seemed to be composed mainly of such giant cells as for instance the small lesion illustrated in Fig. 16. In others there was some fibre formation leading to the appearance of fibromatous regions in some of the tumours (Fig. 10). The vaginal and cervical epithelium over these lesions was usually hyperplastic and showed numerous projections (Fig. 12). With the growth of the sarcomas the epithelium was stretched and ulceration followed.

The early effects of DMBA-painting on the vaginal stroma were not striking. Apart from occasional slight inflammatory changes no generalised effect could be detected. Tumour formation was a late effect and focal in that it arose in some perivascular infiltrates. The thickness of the vaginal as of the uterine stroma was unaffected by the treatment (Fig. 3).

Treatment with DMBA thus causes the occurrence of carcinomas and warts in the vulva, of sarcomas in the vaginal stroma, some hyperplasia of the vaginal epithelium with the rare occurrence of a papilloma and of a carcinoma in this region. The uterine stroma is not affected by the painting. In some rats subjected to prolonged treatment with DMBA squamous metaplasia of endometrial glands and epithelium was observed.

(d) *DMBA-painting of castrated rats* was followed as in virgin rats by marked thickening of both the vulval and vaginal epithelium. While the hyperplasia in the vulva equalled that in virgin rats (Fig. 1), that of the vaginal epithelium was less pronounced than in virgin rats as shown by the total cell counts and the height of the epithelium (Fig. 2). In addition there were fewer downward projections of the epithelium in the treated castrates than in the treated virgin rats.

The differential effect of DMBA painting on the vaginal and the vulval epithelium is indicated by the following figures : the increase in total number of cells was only 115 per cent in the vagina as against 345 per cent in the vulva and the height of the vaginal epithelium was increased to 160 per cent as against 900 per cent in the vulva.

No epithelial tumours whether benign or malignant were found in the vagina during the experiment, while 11 of 13 rats at risk had tumours of the vulva.

All these tumours were carcinomas and were found in animals killed 344 to 406 days after the beginning of treatment. As Fig. 4 shows the vulval tumours tended to appear slightly later than in the virgin rats. Whether this difference is real remains open to doubt since the virgin rats were killed earlier because of the appearance of sarcomas in the vagina.

Fig. 4.

Fig. 5.

Fig. 4.—The percentage of tumours of the vulva after DMBA-painting in castrated rats (B), in castrated rats given oestradiol (C) and in virgin rats (E).

Fig. 5.—The incidence of sarcomas of the vagina after DMBA-painting in castrated rats (B), in castrated rats given oestradiol (C) and in virgin rats (E), and of pre-sarcomatous lesions (— — —) in castrated rats given oestradiol.

Only 3 of the 13 rats at risk developed sarcomas in the vaginal stroma, the first of which was found after 260 days. In another animal there was a tumour of the adrenal and the uterus and cervix showed signs of oestrogen stimulation. The tumours were fibrosarcomas similar to those observed in the virgin animals.

No significant effect of DMBA painting on the width of the vaginal or uterine stroma could be found (Fig. 3). The vaginal stroma tended to be rather thin, with few cells and somewhat hyalinised.

Treatment of castrated rats with DMBA is followed by the appearance of carcinomas of the vulva, of a few sarcomas in the vaginal stroma and by hyperplasia of the vaginal epithelium.

(e) *DMBA painting of castrate rats given oestradiol* is followed by marked thickening of both the vulval and vaginal epithelium (Fig. 1, 2). This was more pronounced in the vulva than in the vagina, the increase in total number of cells for the vagina being 122 per cent and for the vulva 290 per cent. The increase in height of the epithelium was 198 per cent for the vagina and 670 per cent for the vulva. The smaller increase in total number of cells than in height of the epithelium is again accounted for by the increase in cell size. The vaginal hyperplasia in this group was almost as great as in the group of virgin rats, but no papillomas or carcinomas resulted in the vagina.

Of 16 rats at risk 13 had tumours at the vulva of which 9 were carcinomas and 4 warts. Three of the 4 warts and 3 of the 9 carcinomas were found in animals killed before the 350th day.

The width of the vaginal stroma and that of the uterine stroma equalled that of the virgin animals (Fig. 3) whether treated with acetone or with DMBA. Since in the latter no difference between acetone and DMBA-treated rats was found, the increase in width of the stroma of these castrated animals must be attributed to the oestradiol treatment rather than to the DMBA painting.

As in the virgin rats there was some perivascular infiltration in the vaginal stroma after DMBA application and from these lesions or associated with them sarcomas developed in 5 of the 16 rats at risk. The first of these tumours was found in a rat killed after 218 days. The other tumours developed more slowly than in the virgin rats treated with DMBA (Fig. 5).

In addition to these sarcomas, 3 rats had lesions in the vaginal stroma consisting of perivascular infiltrations of large cells resembling those found in early sarcomas and surrounded by some fibre-forming fibroblasts (Fig. 17–19). These lesions may represent a pre-sarcomatous state in that they have some of the characteristics of the early sarcomas but lack evidence of rapid proliferation and are less cellular than sarcomas. The incidence of these lesions which were not found in either the castrated or the virgin rats treated with DMBA is indicated by the broken line in Fig. 5.

Thus the combined treatment of castrates with oestradiol and DMBA is followed by the appearance of carcinomas of the vulva, of some sarcomas and some presarcomatous lesions in the vaginal stroma and by hyperplasia of the vaginal epithelium. The incidence of vaginal sarcomas and the extent of hyperplasia of the vaginal epithelium is intermediate between the DMBA-treated castrate and virgin rats.

### DISCUSSION

In mice (Pan and Gardner, 1948*a*, 1948*b* ; Gardner, 1953 ; Murphy, 1953 ; *Reagan et al.*, 1955 ; von Haam and Scarpelli, 1955) as well as in rats (McEuen, 1938 ; Pfeiffer, 1949 ; Vellios and Griffin, 1957) local application of carcinogens or treatment with oestrogens has led to the appearance of carcinomas of the vagina or cervix rather than of sarcomas. Only Pan and Gardner (1948*b*) observed in mice an almost equal incidence of carcinomas and sarcomas in uterine cervices which were grafted with methylcholanthrene crystals into litter mates. In their experiments the carcinomas appeared earlier than the sarcomas. They observed that the carcinomas grew at a fairly uniform rate while the sarcomas tended to grow more abruptly and progressively. They do not comment on the site of origin of the sarcomas.

In our experiments the sarcomas of the vagina greatly exceeded the carcinomas even in virgin rats where the proportion of vaginal carcinomas to sarcomas was 1 : 16 or 2 : 16 if the vaginal papilloma is included. Furthermore the papilloma and the carcinoma of the vagina appeared later than the sarcomas. On the vulva, on the other hand, warts and carcinomas appeared early—the first warts being noticed by about 200 days—and no sarcomas were found there in these experiments.

The difference in tumour production by the vaginal and vulval epithelium (Table I) is correlated with the degree of hyperplasia induced by painting. While the total number of cells per unit length of the vagina rises to only 115–124 per cent that of the vulva increases to 290–345 per cent and the difference in the increase in the height of the epithelium is even greater : 146–198 per cent for the vagina and 670–900 per cent for the vulva. Furthermore in the vagina the hyperplasia is more marked in the virgin rats than in either the castrate or the castrated rats receiving oestradiol ; in the virgin rats only, are found a carcinoma and a papilloma though the other two groups were exposed to risk for a longer period. These findings suggest (1) a correlation between the induced hyperplasia and

---

### EXPLANATION OF PLATES

FIG. 6.—The vagina of a virgin rat 10 months after acetone painting. The epithelium shows marked cornification and a well-developed stratum granulosum covered by fairly thick layers of keratin. (H. & E.) × 65.

FIG. 7.—A transverse section through a uterine horn of a virgin rat 13 months after acetone painting of the vagina. (H. & E.) × 26.

FIG. 8.—A transverse section through a uterine horn in a castrated rat 13 months after DMBA painting of the vagina. Note the marked reduction in width of the stroma as compared with Fig. 7. (H. & E.) × 26.

FIG. 9.—A transverse section through a uterine horn 9 months after DMBA painting of the vagina in a castrated rat given oestradiol. The width of the stroma is equal to that of the vigin rat in Fig. 7. (H. & E.). × 30.

FIG. 10.—The vagina of a virgin rat painted with DMBA for 13 months shows a papilloma and a sarcoma with marked fibre formation. (H. & E.) × 19.

FIG. 11.—A squamous-cell carcinoma in the vagina of a virgin rat painted with DMBA for 13 months. (H. & E.) × 32.

FIG. 12.—The vagina of a virgin rat painted with DMBA for 10 months shows downward projections of the hyperplastic epithelium and a large cellular fibrosarcoma with numerous multinucleate giant cells. (H. & E.) × 32.

FIG. 13.—An area in Fig. 12 at higher magnification illustrates giant cells with large, often multiple and hyperchromatic nuclei and some abnormal mitotic figures. (H. & E.) × 150.

FIG. 14.—An early sarcoma associated with a perivascular infiltration in the vagina of a virgin rat painted with DMBA for 10 months. (H. & E.) × 67.

FIG. 15.—The perivascular infiltration in Fig. 14 at higher magnification with the characteristic large cells with hyperchromatic nuclei. (H. & E.) × 520.

FIG. 16.—The vagina of a virgin rat painted with DMBA for 12 months showing a small sarcomatous lesion composed mainly of giant cells with large prominent nuclei. (H. & E.) × 210.

FIG. 17.—The vagina of a castrated rat given oestradiol and painted with DMBA for 12 months showing the extent of a " pre-sarcomatous " lesion associated with a perivascular infiltration. (H. & E.) × 33.

FIG. 18.—An area in Fig. 17 at higher magnification to show the perivascular infiltration and the " pre-sarcomatous"' lesion consisting of large cells with hyperchromatic nuclei and associated fibroblasts in thickened connective tissue. (H. & E.) × 90.

FIG. 19.—The large cells of the " pre-sarcomatous " lesion of Fig. 17 and 18 are shown at higher magnification. The large cells with prominent nuclei and coarse chromatin granules resemble those seen in the sarcomas (Fig. 13, 15 and 17). (H. & E.) × 245.

6

7

8

9

Glucksmann and Cherry.

10

11

12

13

14

15

Glucksmann and Cherry.

17

16

18

19

Glucksmann and Cherry.

subsequent tumour formation and (2) an influence of ovarian activity on the hyperplasia of the vagina following DMBA painting.

TABLE 1

| Experiment | Castration | Treatment with | | | Number of rats | | |
|---|---|---|---|---|---|---|---|
| | | | | | | With tumours in | |
| | | Acetone | DMBA | Oestradiol | At risk | Vagina | Vulva |
| A | + | + | − | − | 8 | 0 | 0 |
| B | + | − | + | − | 13 | 3 | 11 |
| C | + | − | + | + | 16 | 5 (+3)* | 13 |
| D | − | + | − | − | 12 | 0 | 0 |
| E | − | − | + | − | 22 | 18† | 21 |

* 3 pre-sarcomatous lesions.
† 16 sarcomas, 1 carcinoma and 1 papilloma.

The difference in the reaction of the vaginal and vulval epithelium may be due to dosage of DMBA, to the existence of a mucous barrier in the vagina, to a greater toxic effect of the painting on the vagina or to an inherent difference in the reactivity of the vaginal and vulval epithelium. Of these various possibilities the mucous barrier might be excluded in view of the fact that sarcomas appeared in the vaginal stroma and that therefore the hydrocarbon has probably penetrated the epithelium in quantities sufficient for tumour induction. We have found little evidence of ulceration of the vagina during the experiment and are thus inclined to attach little weight to the toxic effect of the hydrocarbon on the vaginal epithelium : i.e. that the epithelium was destroyed by the hydrocarbon which passing through to the naked stroma caused sarcomas in this localisation. Since a carcinoma and a papilloma have been induced in the vagina, the epithelium is not entirely refractory to the hydrocarbon and the mucous barrier is not sufficient to protect the vagina. The actual difference in dosage in the vaginal and vulval epithelium is difficult to assess owing to the differences in surface properties of the haired skin of the vulva and the cornified surface of the vagina. We incline to the view that the difference in carcinoma induction in the vagina and vulva is due to differences in the reactivity of the two tissues. The presence of hair follicles in the vulva may contribute to the greater sensitivity of the vulva since a number of the tumours arise in the follicles (Wolbach, 1951).

The induction of sarcomas in the vagina is obviously influenced by the presence and activity of the ovary (Table I). The incidence of sarcomas in castrated rats is significantly lower than in virgin animals painted with DMBA, and the tumours appear later. The presence of an adrenal tumour in one of the castrated rats with a vaginal sarcoma suggests that adrenal corticoids may play some role in the induction of vaginal sarcomas in the castrate or that in this animal at least there was oestrogenic stimulation. The incidence of vaginal sarcomas is increased by oestradiol treatment but not to the level in the virgin rats. The oestradiol was applied twice weekly in order to have variations in the oestrogen levels comparable to the normal oestrus cycle. The effect of this treatment on the width of the uterine and the vaginal stroma shows that the dose level was similar to that of virgin rats though it was probably not quite sufficient for the stimulation of the vaginal epithelium.

The results suggest that oestradiol is not the, or not the only, ovarian hormone which influences the sarcoma production in the vagina as shown by the difference between the castrated and virgin rats. The oestradiol treatment produced some presarcomatous lesions in the vaginal stroma on painting with DMBA in the castrated rats, but whether these lesions are reversible or whether they progress to invasiveness, remains to be investigated. The fact that they occur only in this group (Table I), suggests that oestradiol treatment helps to initiate the carcinogenic process in combination with the DMBA-painting, but that the " promoting " action which occurs in the virgin rats and probably quickly converts the presarcomatous lesion into a sarcomatous one is missing. The observation by Pan and Gardner (1948*b*) that the sarcomas start to grow suddenly and progressively, may be taken as indication why the presarcomatous lesions are not observed in the virgin rats after DMBA painting.

The median latent period for sarcoma production in virgin rats was 11 months, and 12 months in the castrated rats treated with oestrogen. DMBA-painting of the interscapular skin of normal rats induced carcinomas after a median latent period of 9 months and sarcomas after 11 months (Boag and Glucksmann, 1956). The incidence of carcinomas of the skin was also more numerous than that of sarcomas : 73 per cent of all tumours being carcinomas and only 27 per cent sarcomas. In the present experiment sarcoma induction in the vaginal stroma of virgin rats is of the same order of duration as for the skin, but the carcinoma induction is almost missing. In the vulva, on the other hand, carcinomas only and no sarcomas were induced. Whether the differences in types of tumours arising after DMBA-painting at different sites is due to difference in effective dosage, i.e. to differential dilution and absorption of the carcinogen, or to inherent differences in the responsiveness of the tissue at various sites, remains to be decided.

### SUMMARY

1. The effect of local applications of DMBA on the female genital tract of virgin rats, of castrated rats and of castrated rats injected with oestradiol monobenzoate was investigated.

2. In the control animals repeated applications of acetone caused marked cornification of the vaginal epithelium in both virgin and castrated rats but no tumours developed.

3. The vulva responded to the DMBA treatment with marked hyperplasia and subsequent appearance of papillomas and carcinomas. This result was not affected by the presence or absence of the ovary or by the injection of oestradiol to castrated rats.

4. After DMBA-painting the vaginal epithelium showed marked cornification and some hyperplasia in all the rats though not to the same degree as the vulva. Hyperplasia was greatest in the virgin rats, least in the castrated rats and intermediate in the castrated animals given oestradiol. Only one papilloma and one carcinoma were found in 2 of the virgin rats.

5. The DMBA treatment induced sarcomas of the vaginal stroma in 16 of the 22 virgin rats, in 3 of the 13 castrated rats and in 5 of the 16 castrated rats given oestradiol. In addition 3 rats of the last group had pre-sarcomatous lesions.

6. The histogenesis of sarcomas and their relation to focal perivascular infiltration is described and the influence of the ovary and of oestradiol on the width of the uterine and vaginal stroma and on the induction of sarcomas is discussed.

The authors have pleasure in acknowledging their gratitude to Dr. H. B. Fell, F.R.S. and Dr. F. G. Spear for their constructive criticisms of the manuscript, and to Mr. G. C. Lenney for the photomicrographs and graphs.

### REFERENCES

BOAG, J. W. AND GLUCKSMANN, A.—(1956) " Production of cancers in rats by the local application of $\beta$-rays and of chemical carcinogens ". Contrib. to ' Progress in Radiobiology '. Edinburgh (Oliver and Boyd), p. 476.
GARDNER, W. U.—(1953) " Hormonal aspects of experimental tumorigenesis ", in ' Advances in Cancer Research ', Vol. 1. New York (Academic Press), 173–232.
*Idem* AND PAN, S. C.—(1948) *Cancer Res.*, **8**, 241.
GLUCKSMANN, A. AND CHERRY, C. P.—(1956) *Cancer*, **9**, 971.
VON HAAM, E. AND SCARPELLI, D. G.—(1955) *Cancer Res.*, **15**, 449.
HOCH-LIGETI, C.—(1957) *J. nat. Canc. Inst.*, **18**, 661.
MCEUEN, C. S.—(1938) *Amer. J. Canc.*, **34**, 184.
MURPHY, E. D.—(1953) *Amer. J. Path.*, **29**, 608.
PAN, S. C. AND GARDNER, W. U.—(1948*a*) *Cancer Res.*, **8**, 337.
*Iidem*.—(1948*b*) *Ibid.*, **8**, 613.
PFEIFFER, C. A.—(1949) *Ibid.*, **9**, 277.
REAGAN, J. W., WENTZ, W. B. AND MACHICAO, N.—(1955) *Arch. Path.*, **60**, 451.
VELLIOS, F. AND GRIFFIN, J.—(1957) *Cancer Res.*, **17**, 364.
WOLBACH, S. B.—(1951) *Ann. N.Y. Acad. Sci.*, **53**, 517.


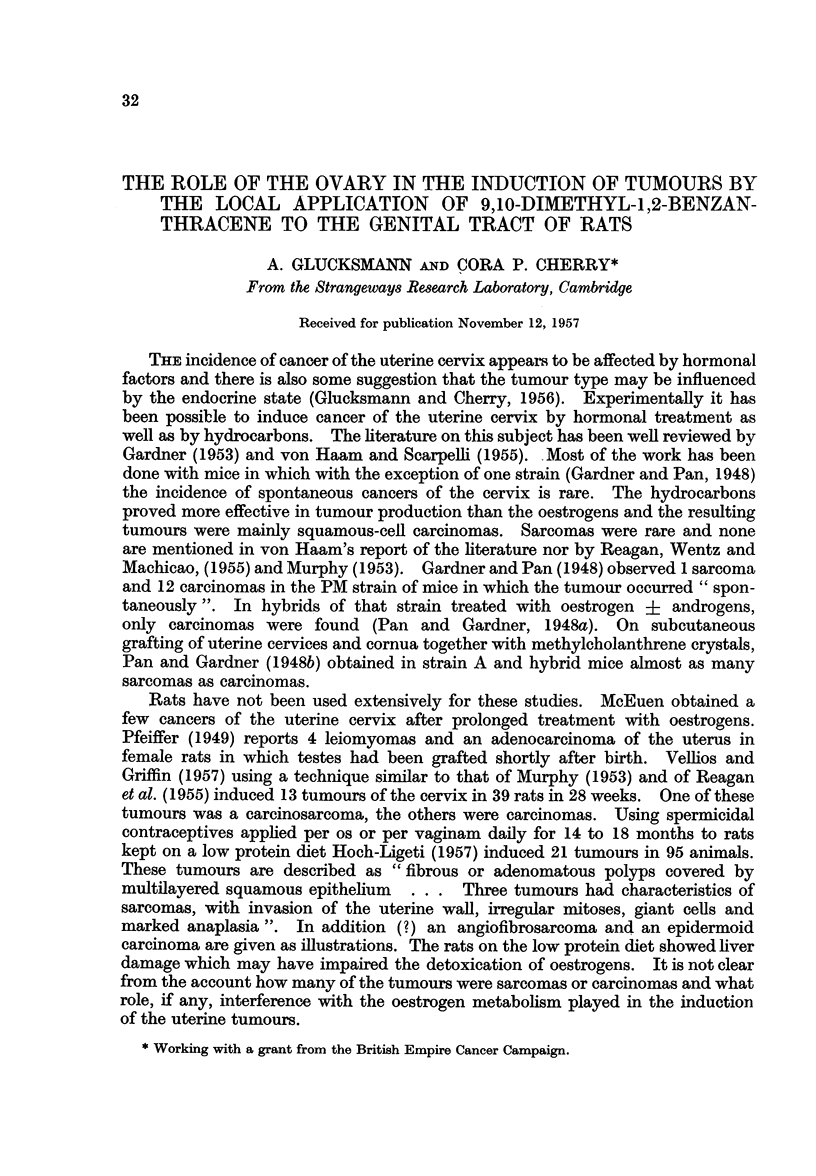

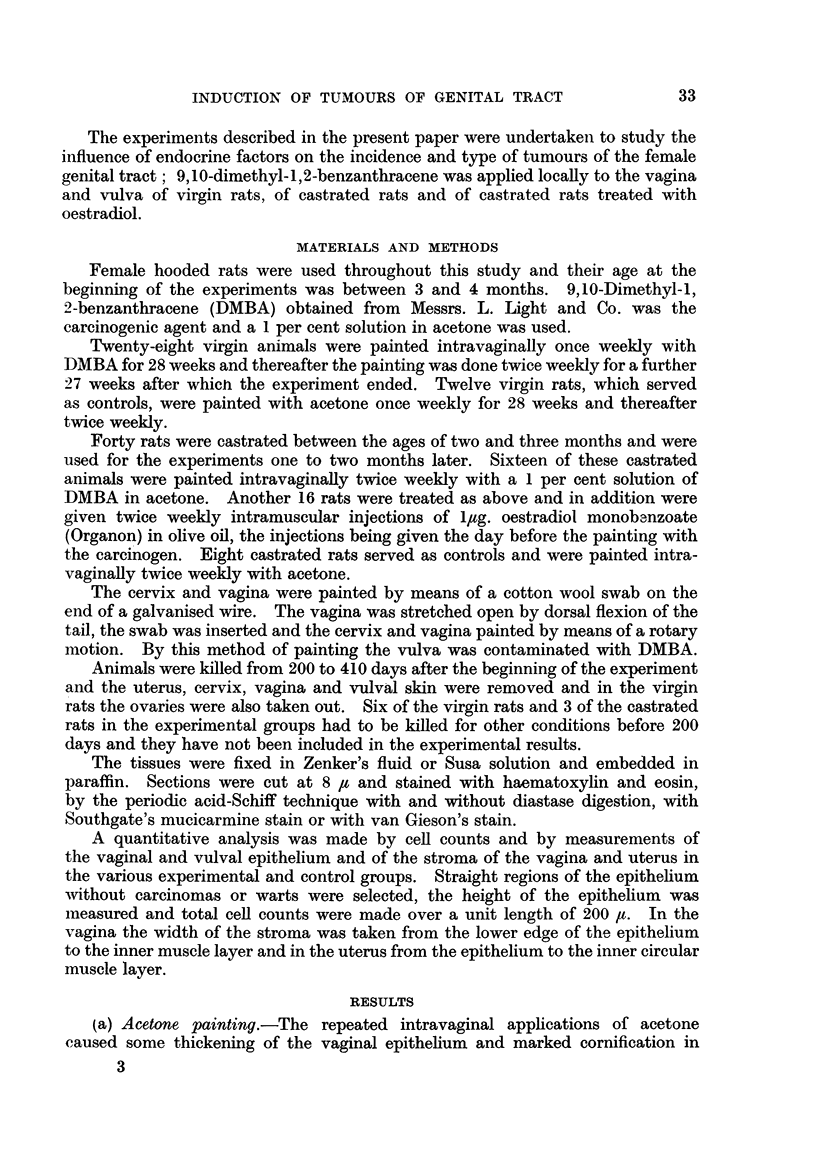

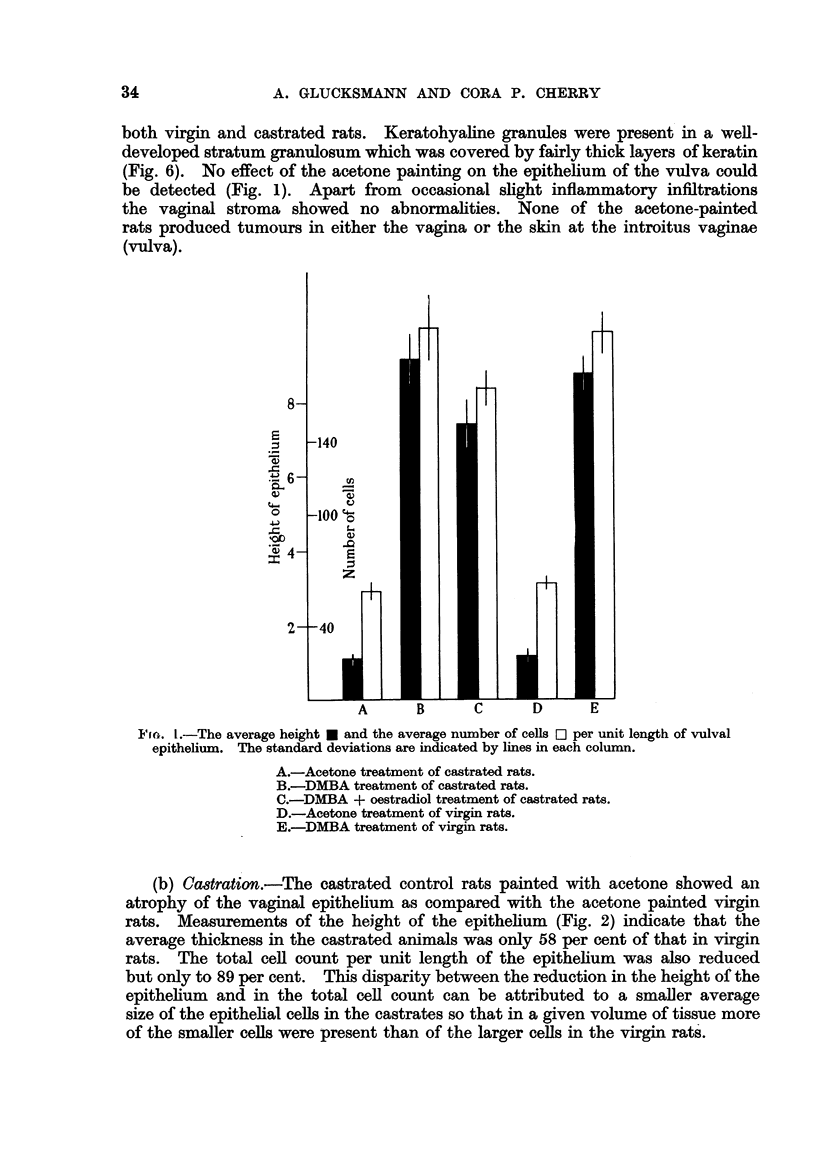

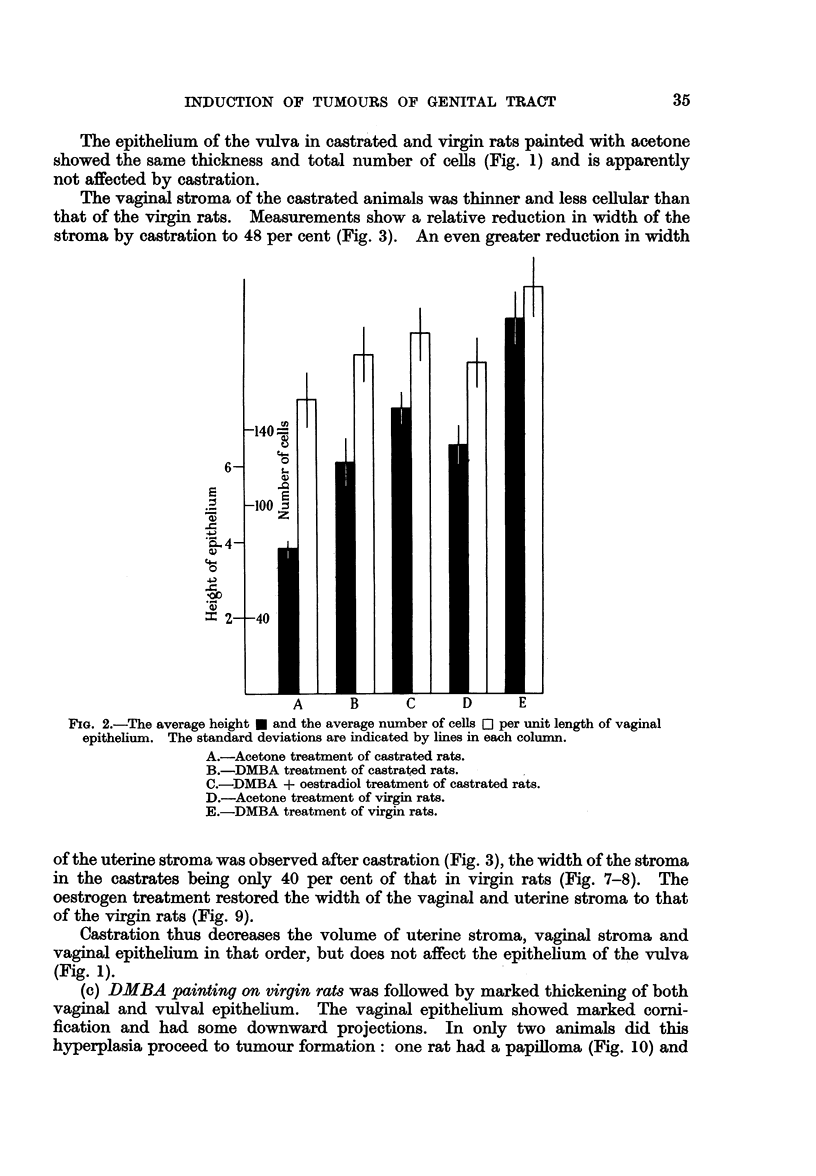

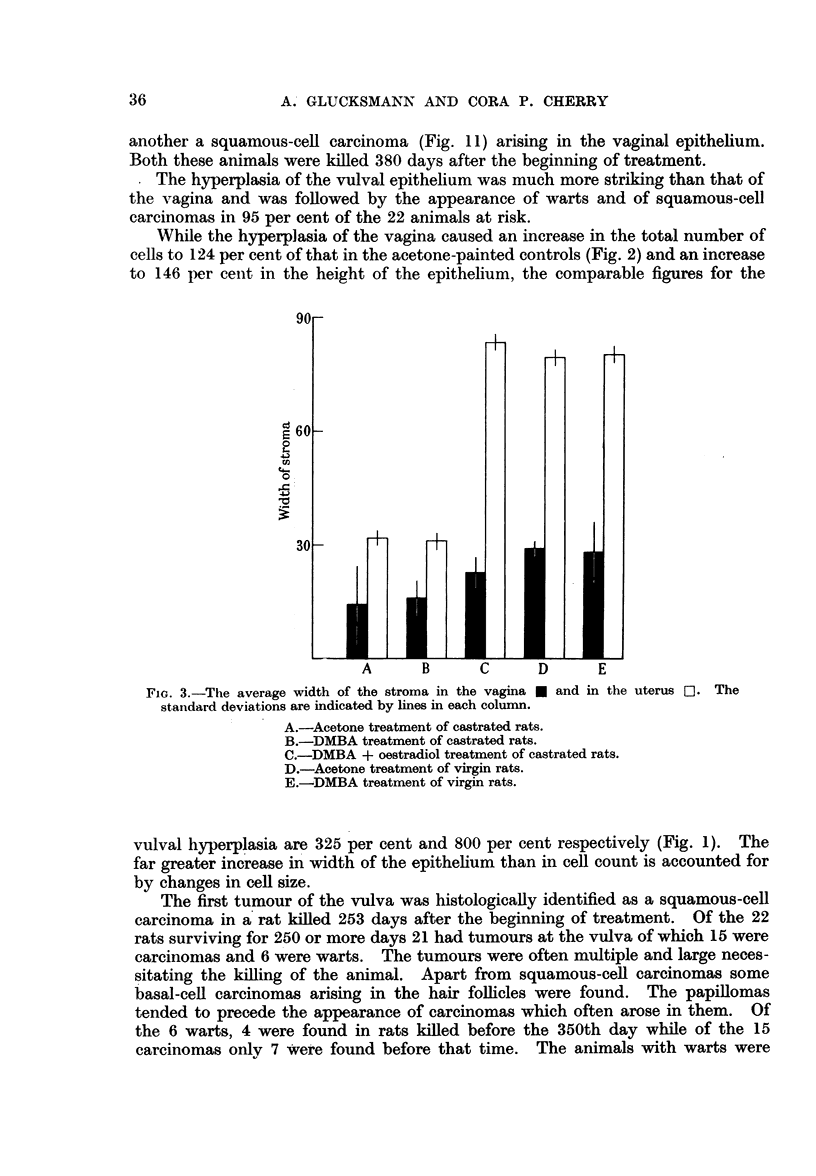

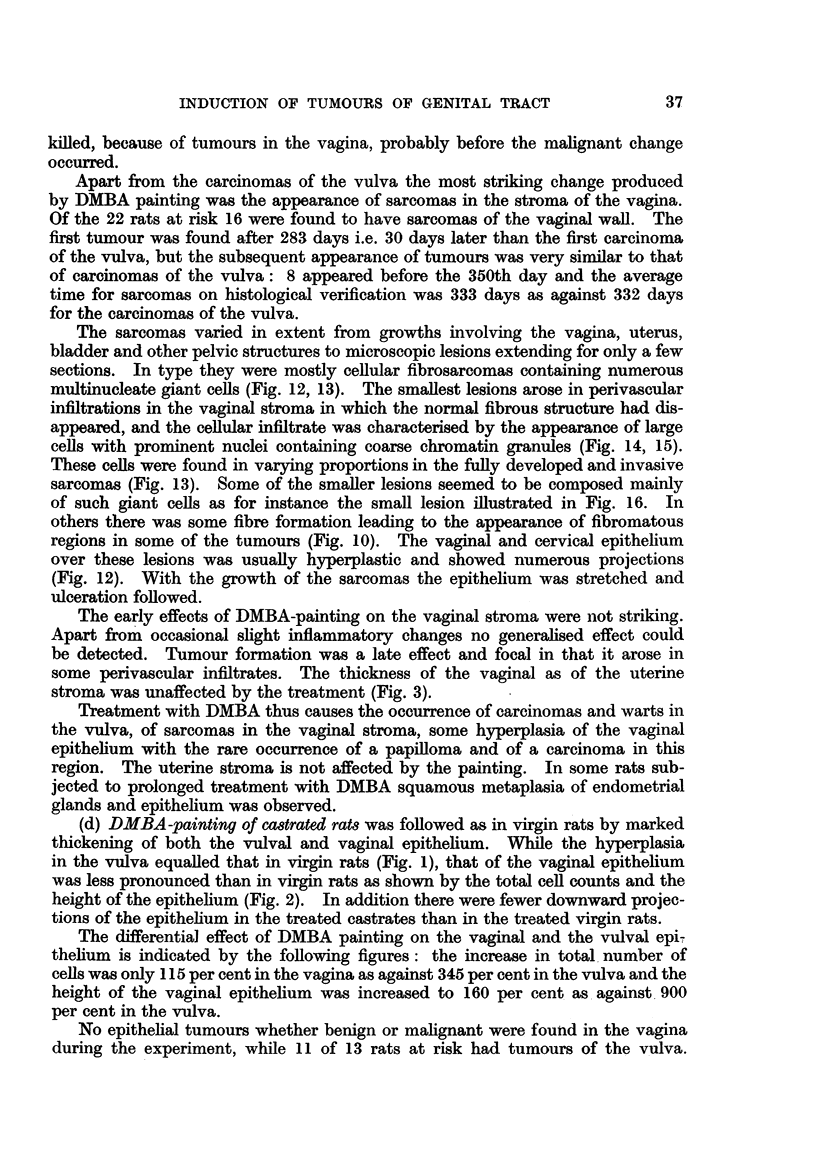

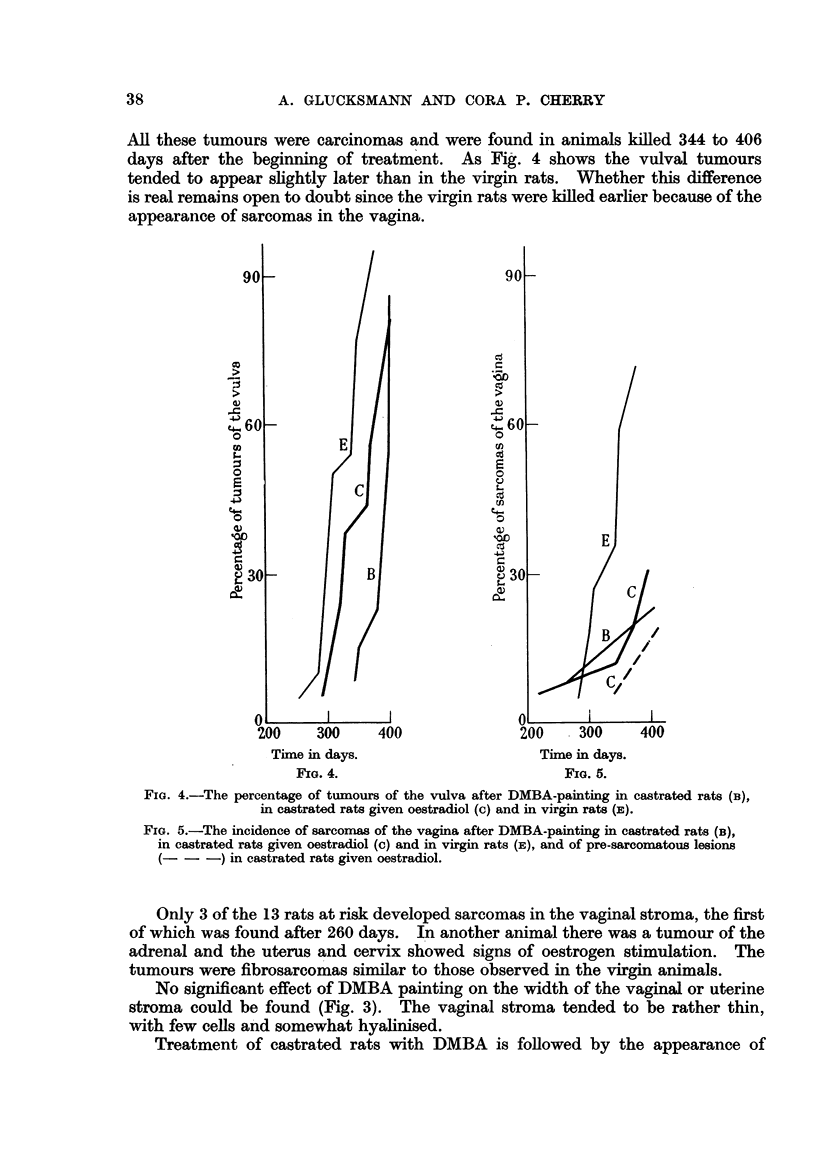

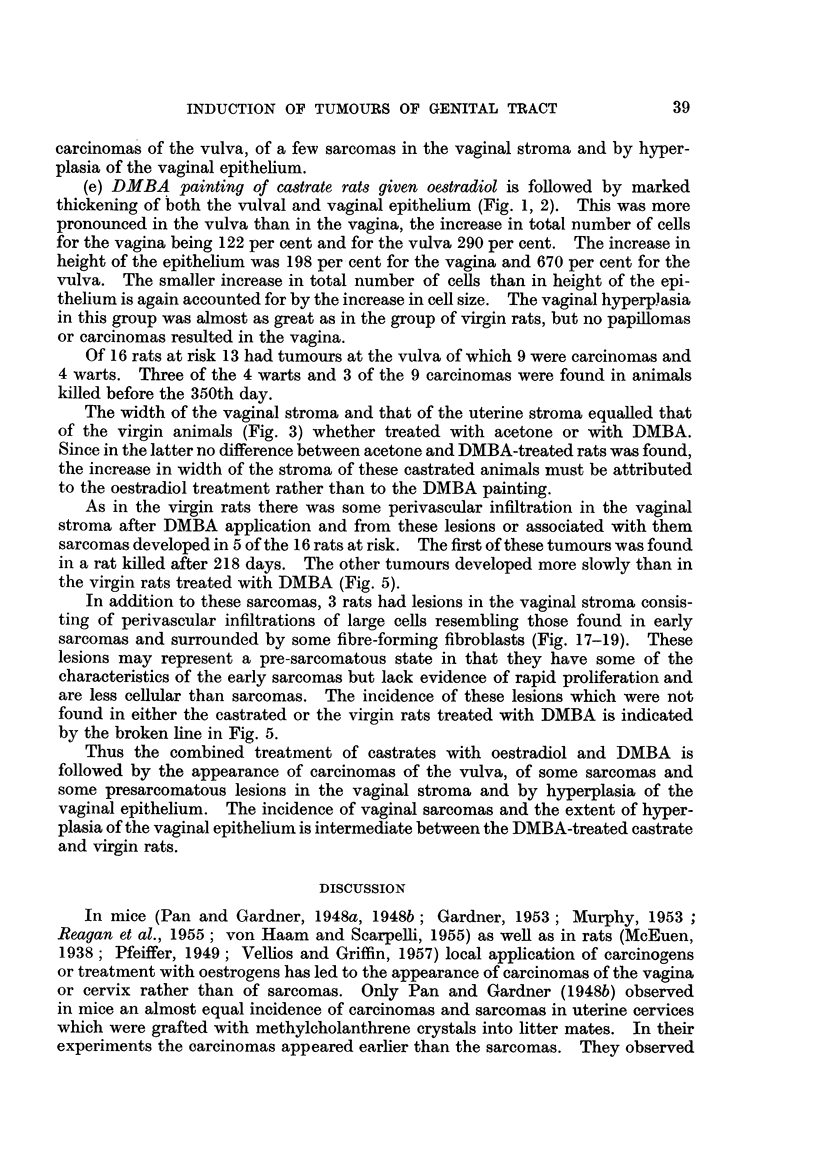

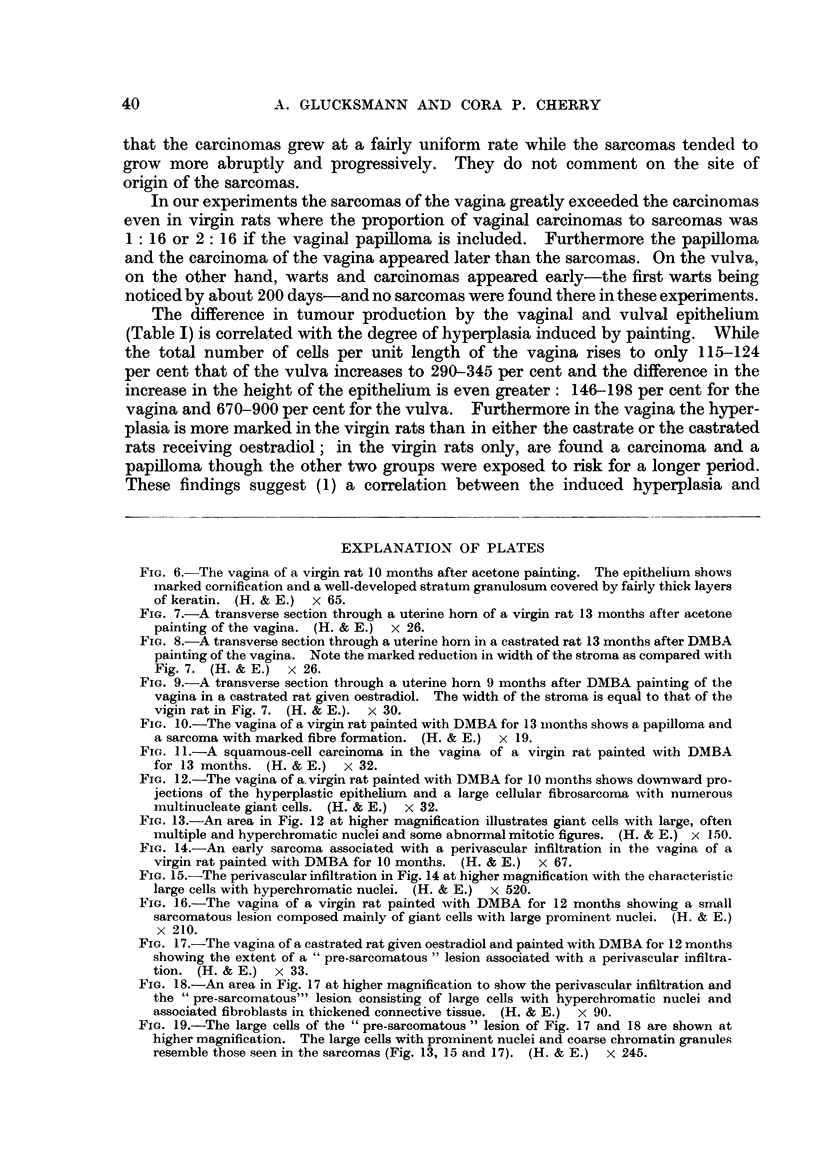

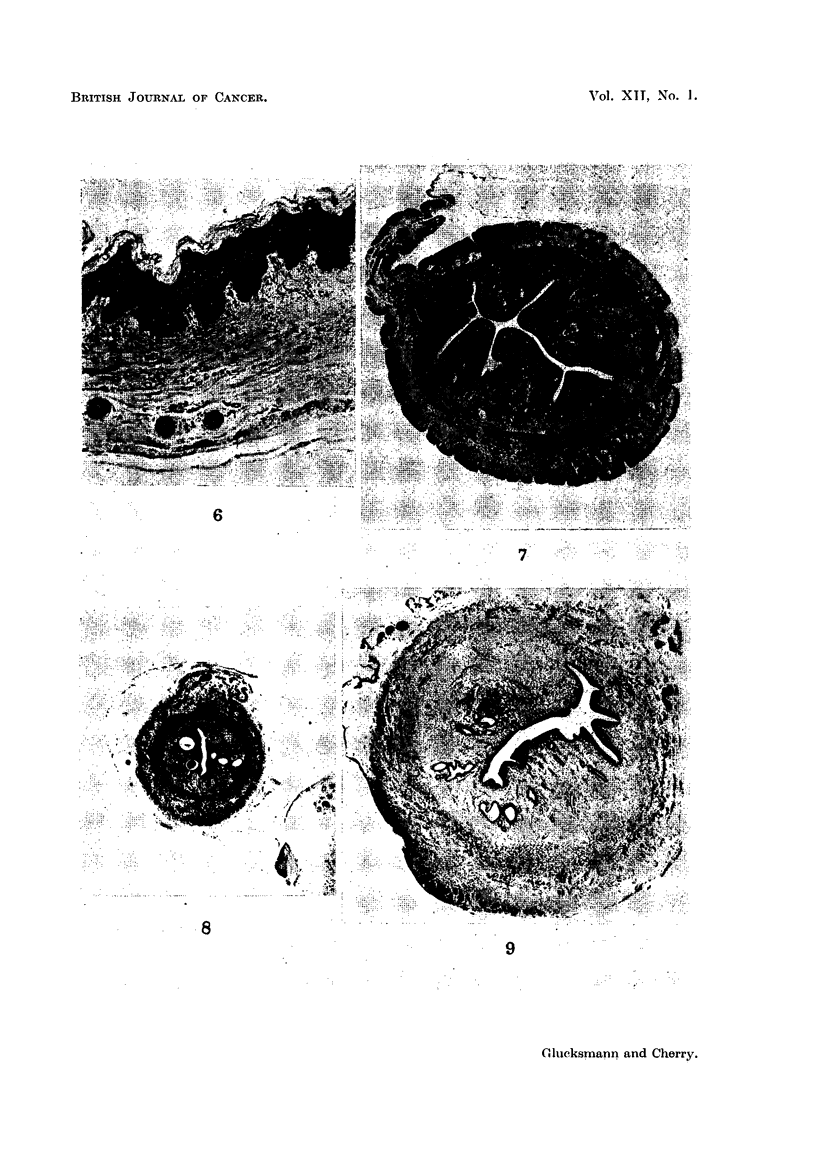

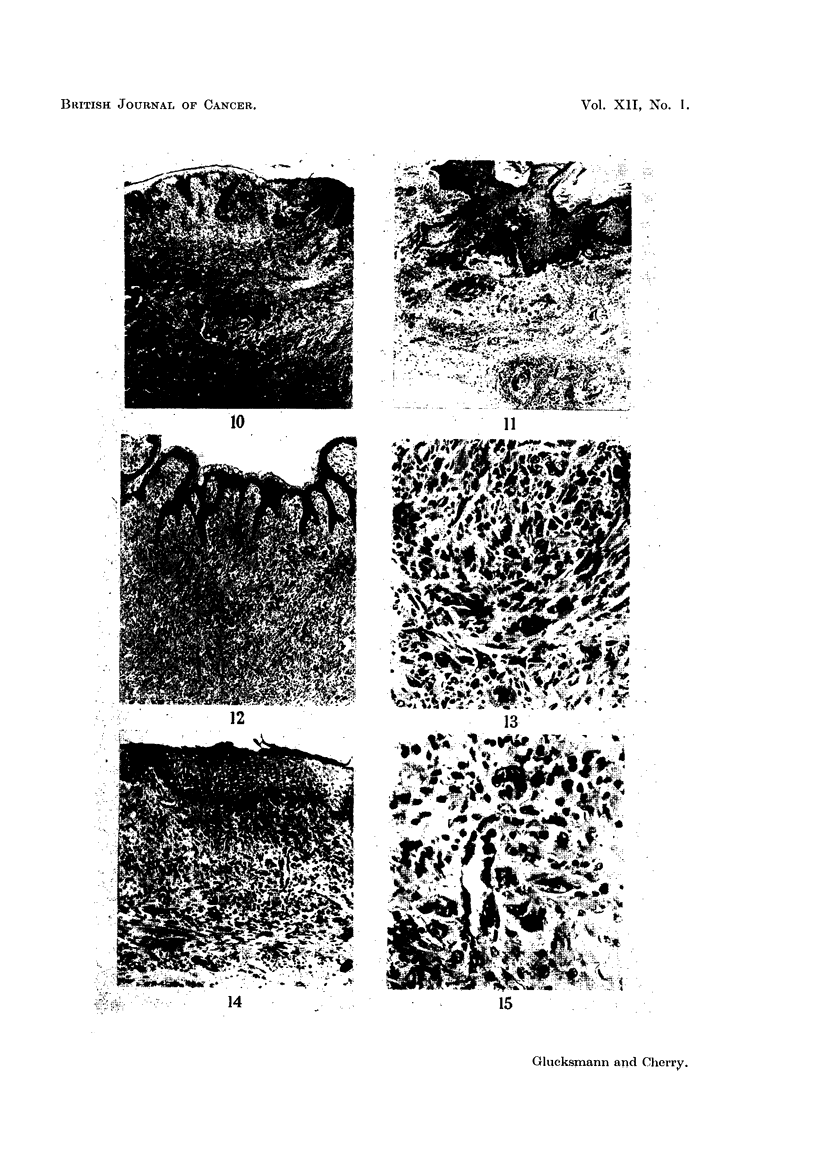

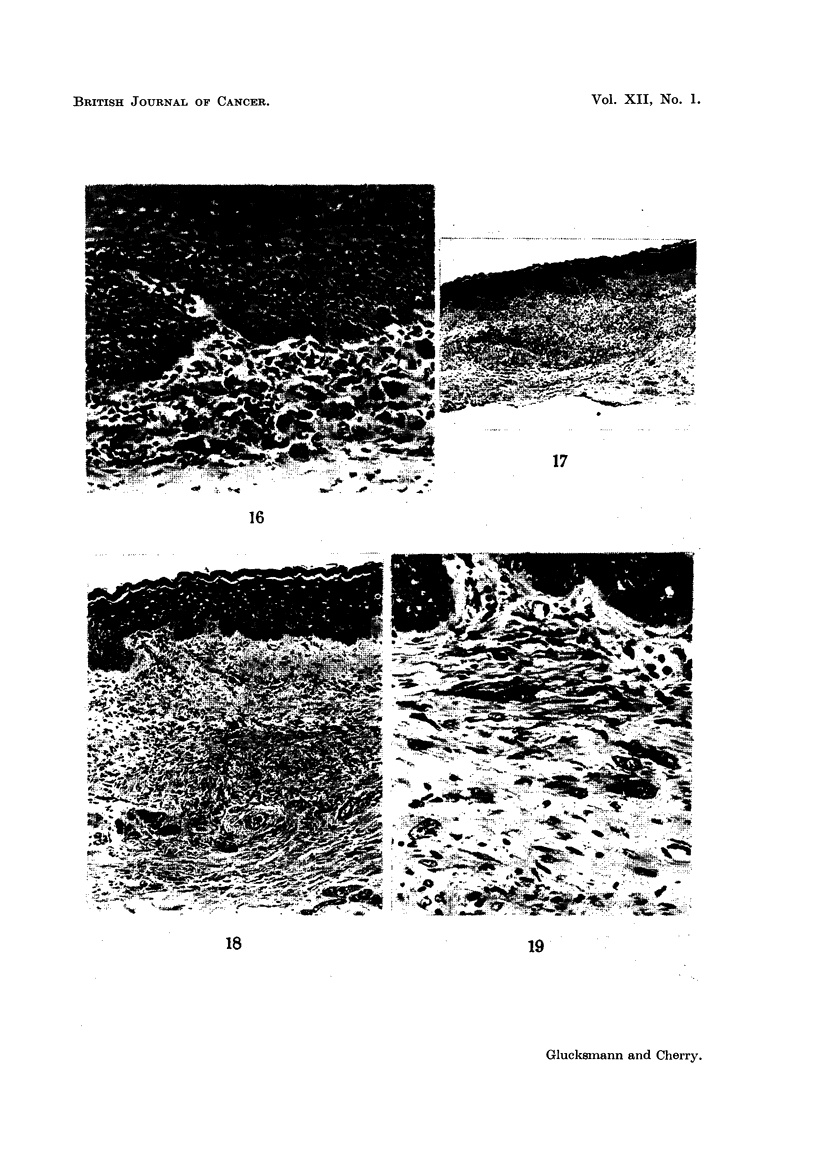

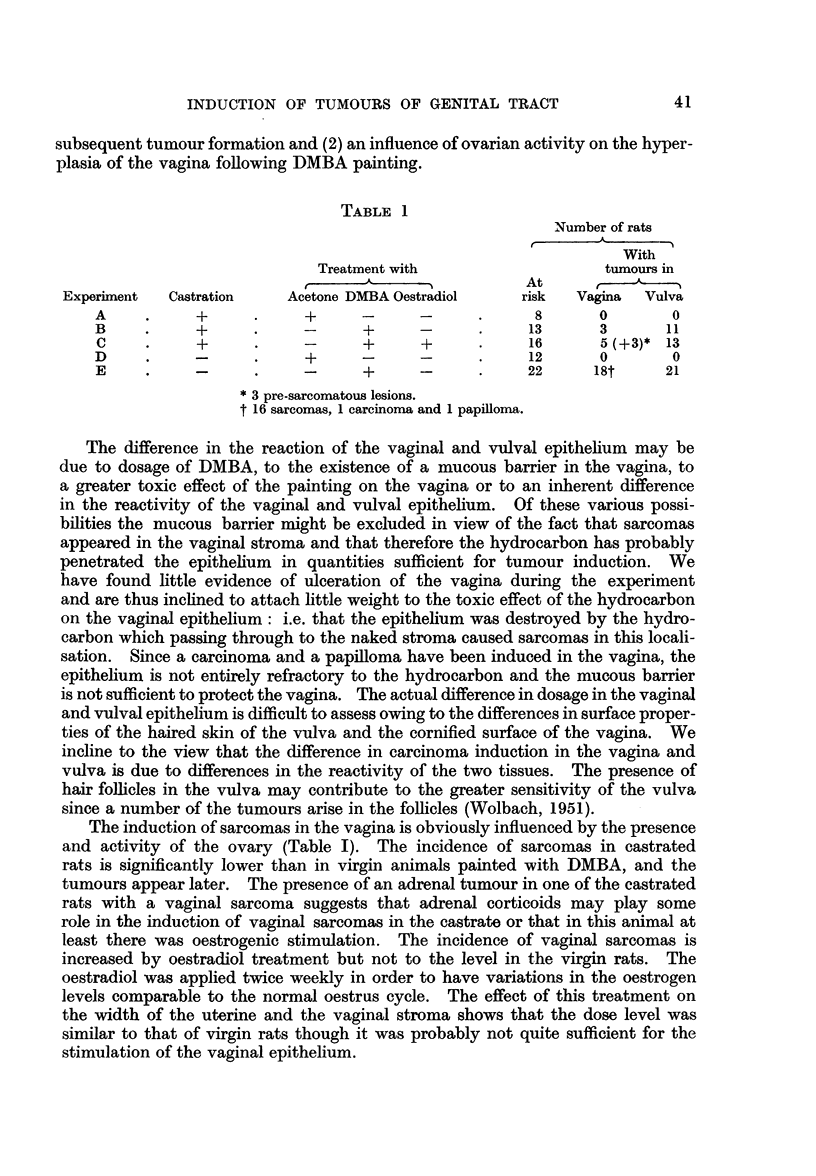

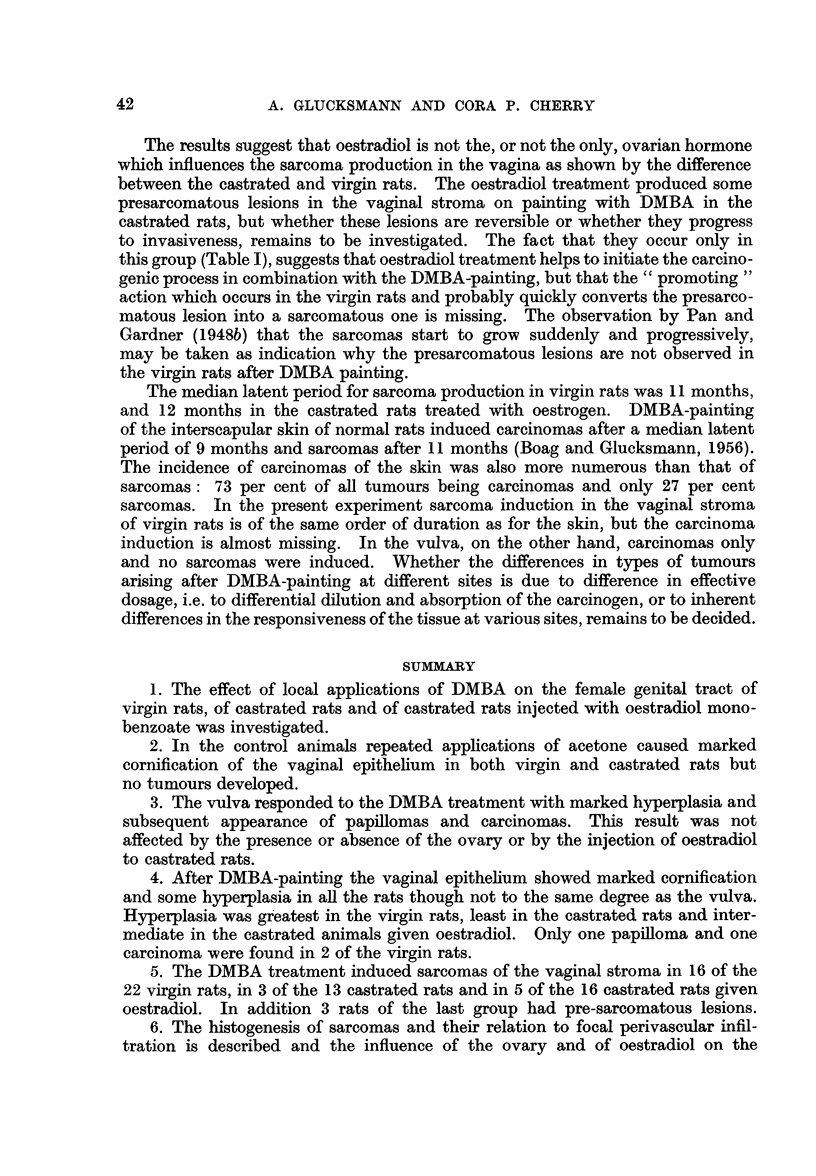

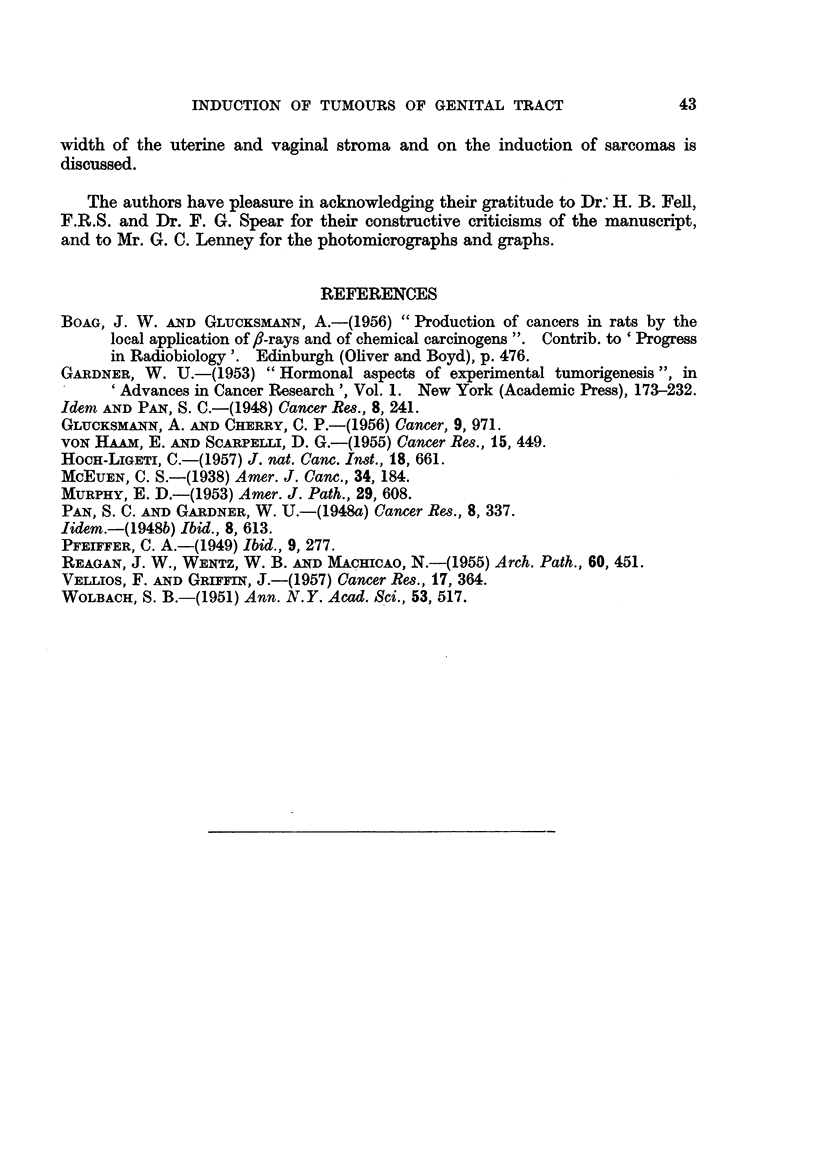


## References

[OCR_00753] CHERRY C. P., GLUCKSMANN A. (1956). Incidence, histology, and response to radiation of mixed carcinomas (adenoacanthomas) of the uterine cervix.. Cancer.

[OCR_00750] GARDNER W. U. (1953). Hormonal aspects of experimental tumorigenesis.. Adv Cancer Res.

[OCR_00756] HOCH-LIGETI C. (1957). Effect of prolonged administration of spermicidal contraceptives on rats kept on low-protein or on full diet.. J Natl Cancer Inst.

[OCR_00757] Mceuen C. S. (1938). Occurrence of cancer in rats treated with oestrone.. Am J Cancer.

[OCR_00765] REAGAN J. W., WENTZ W. B., MACHICAO N. (1955). Induced cancer of the cervix uteri in the mouse.. AMA Arch Pathol.

[OCR_00766] VELLIOS F., GRIFFIN J. (1957). The pathogenesis of dimethylbenzanthracene-induced carcinoma of the cervix of rats.. Cancer Res.

[OCR_00755] VON HAAM E., SCARPELLI D. G. (1955). Experimental carcinoma of the cervix: a comparative cytologic and histologic study.. Cancer Res.

[OCR_00767] WOLBACH S. B. (1951). The hair cycle of the mouse and its importance in the study of sequences of experimental carcinogenesis.. Ann N Y Acad Sci.

